# Biophysical Characterization of α-Synuclein and Rotenone Interaction

**DOI:** 10.3390/biom3030703

**Published:** 2013-09-24

**Authors:** Blanca A. Silva, Ólöf Einarsdóttir, Anthony L. Fink, Vladimir N. Uversky

**Affiliations:** 1 Department of Chemistry and Biochemistry, University of California, 156 High Street, Santa Cruz, CA 95064, USA; E-Mails: blancasilva@hotmail.com (B.A.S.); olof@ucsc.edu (Ó.E.); 2 Department of Molecular Medicine and USF Health Byrd Alzheimer’s Research Institute, Morsani College of Medicine, University of South Florida; 12901 Bruce B. Downs Blvd., MDC 7, Tampa, FL 33612, USA; 3 Institute for Biological Instrumentation, Russian Academy of Sciences, Pushchino 142292, Moscow Region, Russia

**Keywords:** α-Synuclein, Parkinson’s disease, environmental toxin, misfolding, fibrillation, intrinsically disordered protein, pesticide, agrochemical, rotenone

## Abstract

Previous studies revealed that pesticides interact with α-synuclein and accelerate the rate of fibrillation. These results are consistent with the prevailing hypothesis that the direct interaction of α-synuclein with pesticides is one of many suspected factors leading to α-synuclein fibrillation and ultimately to Parkinson’s disease. In this study, the biophysical properties and fibrillation kinetics of α-synuclein in the presence of rotenone were investigated and, more specifically, the effects of rotenone on the early-stage misfolded forms of α-synuclein were considered. The thioflavine T (ThT) fluorescence assay studies provide evidence that early-phase misfolded α-synuclein forms are affected by rotenone and that the fibrillation process is accelerated. Further characterization by attenuated total reflectance Fourier transform infrared spectroscopy (ATR-FTIR) shows that rotenone increases the amount of ordered secondary structure in this intrinsically disordered protein. Morphological characterization by transmission electron microscopy (TEM) and atomic force microscopy (AFM) provide visualization of the differences in the aggregated α-synuclein species developing during the early kinetics of the fibrillation process in the absence and presence of rotenone. We believe that these data provide useful information for a better understanding of the molecular basis of rotenone-induced misfolding and aggregation of α-synuclein.

## 1. Introduction

Parkinson’s disease (PD) is the second most common aging-related neurodegenerative disorder (after Alzheimer’s disease), which is a progressive (degenerative) condition involving a disturbance in the co-ordination of movements. At the onset, the primary clinical symptoms of PD are muscular rigidity, resting tremors, and bradykinesia (slowing of voluntary movement). As PD progresses, other symptoms become more apparent, such as fixed expressionless face, postural instability, akinesia (impaired body movement), depression, and cognitive incompetence [1,2,3]. Despite the extensive research that has led to a better understanding of PD, the etiology of this malady still remains elusive. Among etiologic theories proposed to explain the cause of PD are genetic predisposition, infectious character, toxic exposure, and multifactorial genetic-environmental interactions [4,5,6,7,8,9]. One of the characteristic pathological hallmarks of PD is the loss of dopaminergic neurons in the *substantia nigra pars compacta* (SNpc) region of the brain. The second characteristic feature is the accumulation of proteinaceous intracellular deposits (Lewy bodies, LBs) and neuritic inclusions (Lewy neurites, LNs), which appear in the surviving neurons of the PD-affected brain [4,10,11,12,13,14]. Because LBs and LNs, which consist of granular and filamentous proteinaceous material, are localized in regions where neuronal degeneration takes place in PD patients, PD was classified as a neurodegenerative form of amyloidosis [15,16,17,18]. Amyloidoses constitute a unique group of maladies resulting from an accumulation of misfolded proteins that are deposited intra- or extra-cellularly as insoluble aggregates or insoluble amyloid fibrils. Subsequent immunochemical analysis of LBs and LNs revealed α-synuclein as one of the main components of these PD-related intracellular and neuritic inclusions [10,11,12,13,19,20]. In line with these observations, genetic predisposition was found in familial early onset forms of PD (autosomal dominant PD). Familial PD was first associated with the missense mutation A53T identified in α-synuclein [21]. The genetic predisposition theory was further strengthened by the observation that autosomal dominant early-onset PD is induced in a small number of kindreds as a result of two additional missense mutations in the α-synuclein gene (A30P and E46K) [22,23] or as a result of the hyper-expression of the wild type α-synuclein protein due to its gene triplication [24,25,26].

Several important observations correlating α-synuclein and PD pathogenesis were reviewed in more detail elsewhere [4,15,27,28,29,30,31,32,33,34,35]. Some of these findings are briefly outlined below. A substantial portion of fibrillar material in LBs and LNs was shown to comprise of α-synuclein, and insoluble α-synuclein filaments were recovered from purified LBs [19,36]. The production of wild type α-synuclein in transgenic mice [37] or of WT, A30P, and A53T in transgenic flies [38], leads to motor deficits and neuronal inclusions reminiscent of PD. Under the particular conditions, cells transfected with α-synuclein might develop LB-like inclusions. Numerous studies from different laboratories established that the recombinant α-synuclein easily assembles into amyloid-like fibrils *in vitro* and this process is modulated by familial point mutations [39,40]. α-Synuclein is abnormally phosphorylated, ubiquitinated, and nitrated in pathology-related inclusions. Co-expression of chaperones or β-synuclein with α-synuclein in transgenic animals was shown to suppress the neurodegeneration. α-Synuclein-positive proteinaceous deposits were shown to accumulate in several animal models where Parkinsonism was induced by exposure to different neurotoxicants [41]. The last observation supports the hypothesis that some environmental factors, in particular exposure to pesticides and transition metals such as manganese and iron, may play an important role in events leading to idiopathic forms of PD [41,42,43,44,45,46,47]. In fact, certain neurotoxins were shown to promote and accelerate the development of PD, whereas other environmental agents (such as caffeine and nicotine) possess some neuroprotective properties and may decrease the risk of PD [48,49,50]. Among the PD-promoting environmental toxins are: metals [42,51,52,53,54,55,56]; solvents [57,58,59,60,61]; carbon monoxide [62]; 1-methyl-4-phenyl-1,2,3,6 tetrahydropyridine (MPTP) [6]; and some pesticides and herbicides [63,64,65,66,67,68].

The interaction of α-synuclein with pesticides *in vitro* has been established as a pivotal event triggering misfolding and accelerating fibrillation of this important protein [45,47,69,70]. Therefore, elucidating the mechanism of this interaction is paramount for understanding the properties of α-synuclein-pesticide complexes and the structural features leading to pesticide-promoted misfolding and aggregation of α-synuclein. The presence of partially folded intermediate species as a critical component in the α-synuclein fibrillation process has been reported [40,71,72], and several environmental factors were shown to affect the early stages of α-synuclein fibrillation and modulate formation of the early oligomeric species [73,74,75,76,77,78,79]. This suggested that early oligomeric species may play a significant role in triggering Parkinson’s disease and other protein deposition diseases. Additional studies, focused on the toxic role of early fibrillation intermediates, have supported this hypothesis [80]. For example, Bucciantini *et al*. showed that the toxicity of aggregated species appears to be a result of their high concentration inside the cell, and that the interactions of aggregates with cellular compartments eventually impair normal cell function [80]. Similarly, Walsh *et al*. [4] suggested considering protofibril structures as the intermediate species that directly interact and affect neuronal cells. These precursors to fibrillar structures appear to alter the physiology of cultured neuronal cells. For example, the electrical activity of neuronal rat cells was shown to be affected by the direct interaction with intermediate protofilaments [81]. In bovine serum albumin (BSA) misfolding studies, Mititello *et al*. showed the effect of the net charge of the protein surface on the protein aggregation kinetics [82]. Based on FTIR analysis, it was suggested that misfolding is frequently accompanied by β-sheet formation. In addition, by combining dynamic light scattering with FTIR, the early β-sheets were characterized as being uniformly small [82].

Some pesticides induce α-synuclein misfolding, thereby accelerating the fibrillation process [69,70,83,84]. Most pesticides are hydrophobic in character, and this property may play an important role in triggering α-synuclein aggregation. However, the mechanism of this interaction is unclear and many questions remain unanswered. Is the interaction of monomeric forms of α-synuclein with pesticides critical for fibrillation to take place? Does the interaction of early partially folded α-synuclein with pesticides induce a higher propensity to fibrillate compared to that of the late stage aggregated forms of misfolded α-synuclein? Do pesticides remain bound to α-synuclein once misfolding and fibrillation is triggered? Biophysical studies are essential to address these questions [85,86,87,88,89]. 

The purpose of this study was to characterize the properties of α-synuclein-pesticide complexes, their morphological characteristics, and changes in secondary structure through the early phase of the fibrillation process. The techniques utilized were: ATR-FTIR, the ThT florescence assay, AFM and TEM, all of which are well-established techniques for conducting biophysical studies of aggregating proteins. The major focus was on characterization of the effects of the interaction of α-synuclein with rotenone during the early lag phase of the fibrillation process by ATR-FTIR, followed by typifying the morphological changes through the use of AFM and TEM imaging techniques. 

ATR-FTIR is a well-established form of vibrational spectroscopy ideal for monitoring protein secondary structure under a variety of conditions. For this reason, it was used here to monitor the time course of the secondary structure changes during rotenone interaction with α-synuclein [90,91,92]. The major protein FTIR bands are typically associated with vibrations resulting from the C=O, O–H, −COOH, −COO^−^, and S–H chemical groups. The chemical groups can undergo vibrations characterized as stretching, twisting and rotation. Many of these vibrations are not characteristic of a single type of oscillation, but rather result from a combination of oscillations coupled to neighboring bonds and groups [91,92]. To conduct biophysical protein studies, vibrational spectroscopy exploits three major bands. These bands are referred to as the Amide modes or Amide bands I, II, and III and absorb energy in the 1700–1600, 1600–1500, and 1350–1200 cm^−1^ regions, respectively. 

ATR-FTIR spectroscopy is a unique tool used in biophysical studies of proteins. It offers a wealth of information regarding protein structure, the environment of the amino acid side chains, and ligand binding to proteins, in addition to details about redox states, bond length, bond strength, bond angles, hydrogen bonding and electric field changes. This information is a result of changes in the electron density distribution and conformational freedom arising from the various secondary structures [90,91]. Because the Amide I vibrational band is essentially unaffected by the amino acid side chains, this vibrational band is most commonly used to monitor changes in secondary structure of the protein back-bone. The most common structural changes involve the α-helix, the random coil and the β-sheet [90,91,93]. 

When conducting ATR-FTIR studies, the Amide I band signal is monitored from 1700–1600 cm^−1^. This signal is mainly associated with the C=O stretching vibration resulting from hydrogen bonding and distortion of Amide linkages. Minor contributions have been observed from out-of-phase CN stretching vibrations, CCN deformations, and NH in-plane bends [90,94]. Within the 1700–1600 cm^−1^ spectral region, bands observed at 1660 cm^−1^ or above generally arise from non-hydrogen bonded structures. Bands observed below 1645 cm^−1^ correspond to strongly hydrogen-bonded structures, such as β-sheet. Bands observed within the 1645–1660 cm^−1^ range correspond to weaker hydrogen-bonded structures, and these signals are usually due to α-helices or unordered structure [94]. 

ATR-FTIR spectral bands are commonly analyzed using second-derivative and deconvolution techniques [90,91,94]. These techniques allow the resolution of overlapping structural components within a broad spectral envelope. In addition, monitoring spectral differences under different conditions provides information related to the conformational changes in proteins [92,95].

## 2. Results and Discussion

### 2.1. Kinetics of the α-Synuclein Fibril Formation in the Presence of Rotenone

**Figure 1:**
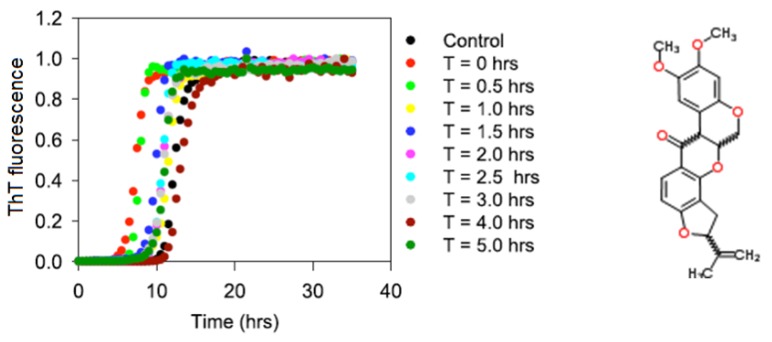
The effects of rotenone on α-synuclein fibrillation. T indicates the time during the fibrillation process at which rotenone was introduced. Chemical formula of rotenone is shown to the right.

The objective of this project was to characterize the interaction of α-synuclein with rotenone during various stages of the fibrillation process. The results are expected to strengthen the epidemiological and clinical evidence that links pesticide exposure with an increased incidence of PD [10,96,97,98,99]. The effects of rotenone on the aggregation and fibrillation kinetics of α-synuclein were monitored by starting the fibrillation process in the absence of rotenone and then introducing the pesticide at selected time points during the fibrillation process using the plate-based and manual assays with the histological ThT dye (see Figure 1). The changes in the ThT fluorescence signal is commonly used to monitor the formation of fibrils since the fluorescence signal enhancement of this dye is associated with the dye binding to the fibrils. 

Figure 1 shows the typical fibril formation kinetics induced by the incubation of 70 μM α-synuclein at pH 7.5 and 37 °C. The fibrillation kinetics in all instances exhibited the characteristic sigmoidal appearance with an initial lag phase, followed by a growth or elongation phase and a final equilibrium phase. These curves are consistent with the nucleation-dependent polymerization model and show that rotenone alters the fibrillation process by decreasing the nucleation phase. The results shown in Figure 1 and Table 1 suggest that monomeric α-synuclein and early small α-synuclein oligomers are more susceptible to rotenone than late-stage larger oligomeric species as evidenced by the acceleration of fibril formation when rotenone was introduced during the early fibrillation phase. These effects are noteworthy because rotenone induces major PD symptoms in rats, and previous molecular studies have demonstrated that rotenone disrupts complex I in mitochondrial respiration [70,100,101,102]. 

**Table 1:** Effects of added rotenone on kinetic parameters of α-synuclein fibrillation.

Rotenone introduction time (h)	Lag time (h)	*k_app_* (h^−1^)
N/A	9.8 ± 1.8	1.2 ± 0.4
0.0	6.9 ± 1.2	1.7 ± 0.3
0.5	7.7 ± 1.0	2.3 ± 0.4
1.0	9.9 ± 2.0	1.7 ± 0.4
1.5	8.8 ± 1.1	1.9 ± 0.2
2.0	9.6 ± 0.4	1.8 ± 0.3
2.5	9.3 ± 0.7	1.9 ± 0.4
3.0	9.2 ± 1.1	1.7 ± 0.3
4.0	10.8 ± 1.0	1.2 ± 0.3
5.0	10.1 ± 0.6	2.1 ± 0.4

The manual ThT assay was conducted in parallel with the in-plate ThT assay, the only difference being that in the manual assay rotenone was introduced at earlier and more frequent time intervals during the fibrillation process; the results of these manual studies are consistent with those from the in-plate ThT assay, and support the noted observations above. Earlier we showed that the hydrophobic character of pesticides is a property that plays a pivotal role in inducing fibrillation [84]. Accordingly, this observation suggests that rotenone interacts preferentially with the hydrophobic regions of α-synuclein. This interaction can induce a conformational change that facilitates aggregation and fibrillation. Previous studies have indicated that the interaction of metals with α-synuclein triggers and accelerates protein aggregation [103]. These findings are supported by the work of Bernstein *et al*. who proposed that metals, such as lead or zinc, alter the native charge state of a protein, inducing α-synuclein misfolding and triggering amyloid formation [104].

### 2.2. Analysis of α-Synuclein-Rotenone Fibrillation by ATR-FTIR

**Figure 2:**
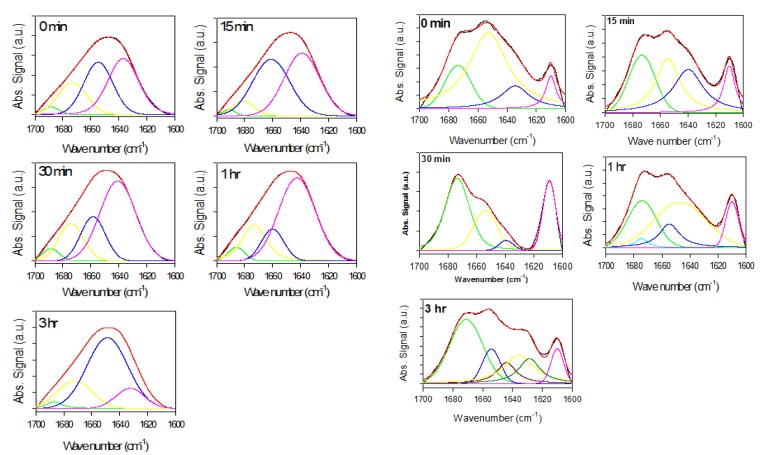
Attenuated total reflectance Fourier transform infrared spectroscopy (ATR-FTIR) spectra of supernatant samples (left panels) and precipitate samples (right panels) taken at time of rotenone addition. Black curves are experimental signals; red curves are fits. Signals under curve represent deconvoluted experimental curve-fit signals bands used to determine structural content in a given sample and positioned at ~1635 cm^−1^ (pink lines), ~1657 cm^−1^ (blue lines), ~1678 cm^−1^ (yellow lines), ~1695 cm^−1^ (green lines).

**Figure 3:**
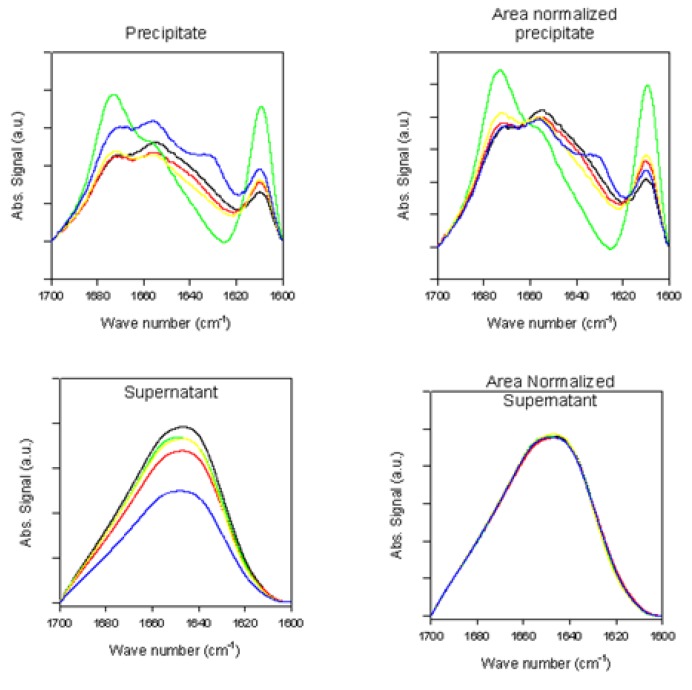
Superimposed ATR-FTIR spectra of precipitate samples (top panels) and supernatant samples (bottom panels) taken at time of rotenone addition (black, red green, yellow and blue curves correspond to measurements taken at 0, 0.25, 0.5, 1, and 3 h, respectively).

**Figure 4:**
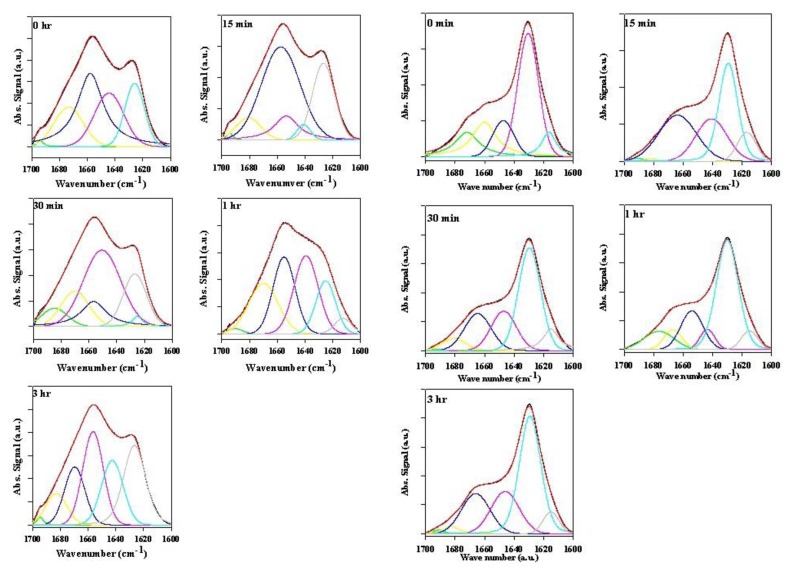
ATR-FTIR spectra of supernatant samples (left panels) and precipitate samples (right panels) taken at the end of the fibrillation process.

**Figure 5:**
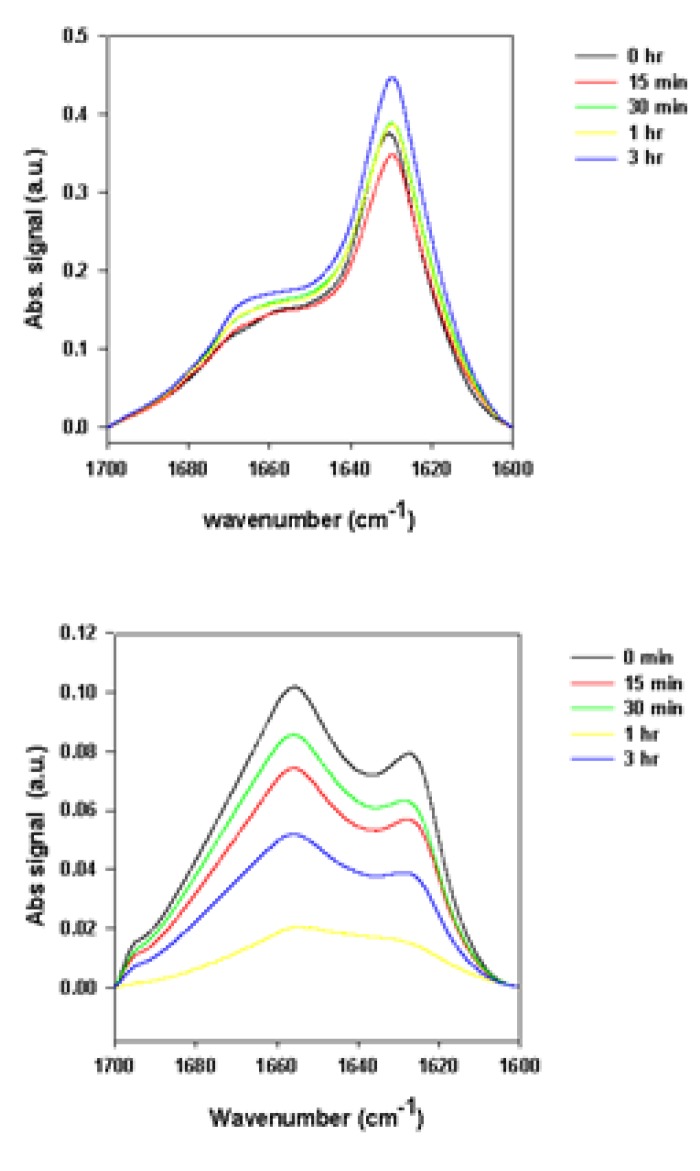
Superimposed ATR-FTIR spectra of precipitate samples (top panels) and supernatant samples (bottom panels) taken at the end of the fibrillation process.

**Figure 6:**
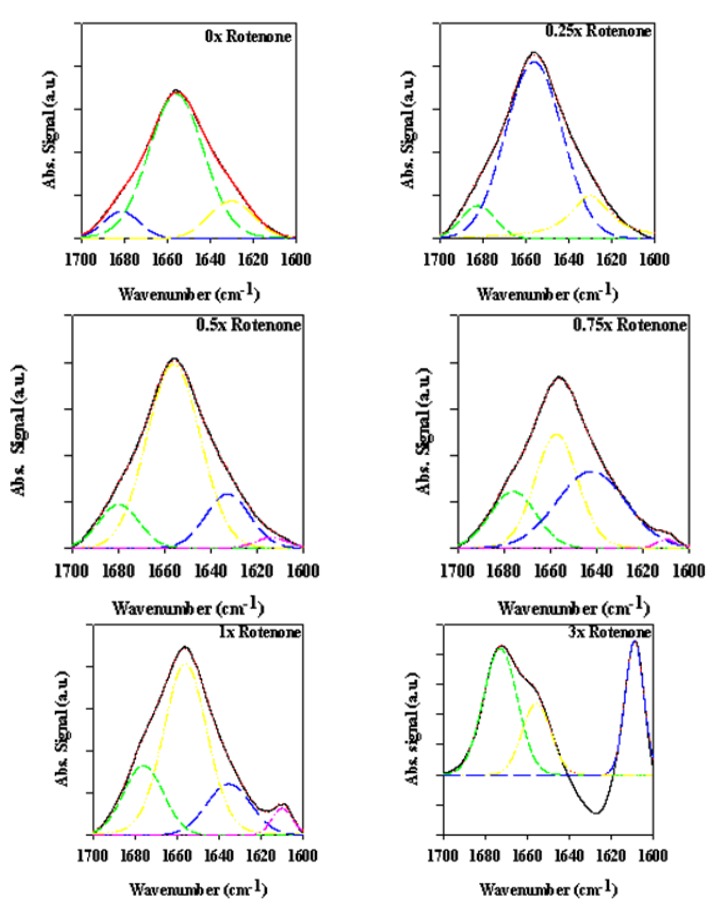
ATR-FTIR spectra of bovine serum albumin (BSA) in the presence of various rotenone equivalents after a 30-min incubation period.

**Figure 7:**
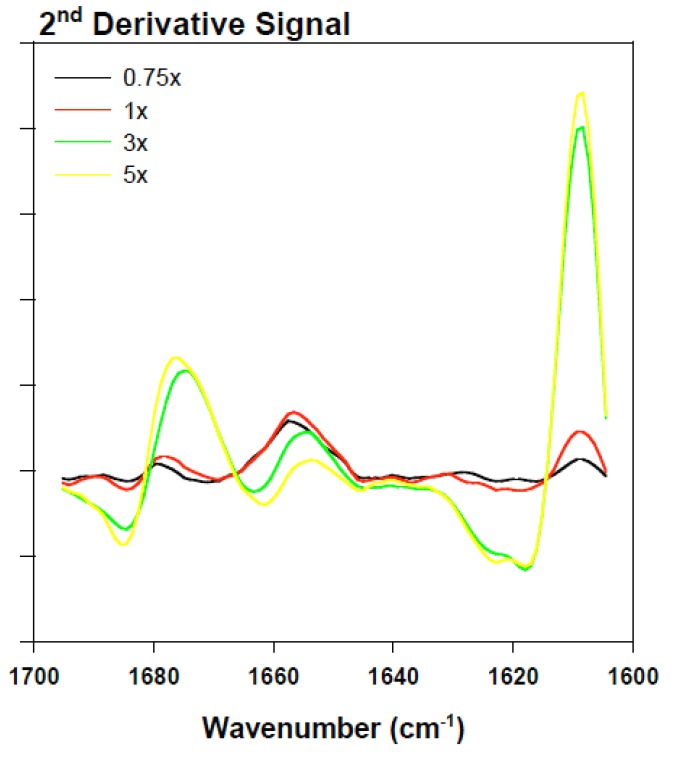
Superimposed inverted BSA second derivative signal in the presence of various rotenone equivalents following a 30-min incubation period.

The plate-based ThT assay demonstrates that rotenone accelerates the fibrillation process of α-synuclein. The rotenone-promoted structural transformations were further characterized by ATR-FTIR. ATR-FTIR spectra in the Amide I region were collected for the supernatant and precipitated samples at various times following rotenone introduction and at the end of the fibrillation process (Figure 2, Figure 3, Figure 4, Figure 5, Figure 6 and Figure 7). The Amide I region is primarily governed by the stretching vibrations of the C=O (70–85%) and C–N groups (10–20%). The exact band position is determined by the backbone conformation and the hydrogen bonding pattern.

#### 2.2.1. Effects of Rotenone on α-Synuclein ATR-FTIR Spectra during the Fibrillation Process

The ATR-FTIR spectra of supernatant and precipitate samples recorded during the fibrillation process are shown in Figure 2, whereas the spectra of the total samples are shown in Figure 3. The supernatant spectra in the absence or presence of rotenone are characteristic of substantially unstructured polypeptide chains (Figure 2 and Figure 3). However, in the presence of rotenone, the spectra of the precipitate sample reflect increased content of ordered secondary structure (Figure 2 and Figure 3), as reflected by the decrease in the FTIR band at 1650 cm^−1^ and prominent changes around 1610 cm^−1^ [91,93]. 

The signal around 1610 cm^−1^ is sharp and relatively weak, but increases over time in the presence of rotenone. This signal is assumed to reflect amino acid side-chain interactions. More specifically, it has been associated with the aromatic side chain of tyrosine [105,106]. At the end of the fibrillation process, a prominent signal at ~1630 cm^−1^ is observed, reflecting increased content of the ordered β-structure [91]. Similar structural changes were observed in previous studies and were attributed to factors that promote self-interaction to yield stable aggregates, pre-fibril and fibril species [40,107,108]. Increasing β-structure content was also observed in the later stages of the fibrillating process. To further analyze the structural differences during the fibrillation process, the spectra of the supernatant samples taken at different times were normalized and superimposed; the precipitate samples were also normalized and superimposed (Figure 3). The supernatant Amide I band during the fibrillation process is consistent with that of mostly unfolded species (Figure 3). With time, the intensity of this band decreased, whereas the spectral shape did not change. This is evident from the fact that upon normalization and superposition of the spectra, they become indistinguishable. This suggests that the overall secondary structural content remains the same, but the amount of available material decreases over time. The superimposed precipitate signals show a loss in intensity at ~1650 cm^−1^ reflecting decreased amount of unordered structure, an increase at ~1610 cm^−1^, ~1620 cm^−1^, and ~1680 cm^−1^, which is associated with the increased content of β-sheet (~1620 cm^−1^ and ~1680 cm^−1^) and/or β-turns (~1680 cm^−1^), or increased contribution of side chains (~1610 cm^−1^).

#### 2.2.2. Effects of Rotenone on α-Synuclein ATR-FTIR Spectra at the End of the Fibrillation Process

Figure 4 and Figure 5 represent the ATR-FTIR spectral analysis of the supernatant and precipitated pools at the end of the fibrillation process in the presence and absence of rotenone. For the precipitate samples, signals within each sample pool are clearly very similar and suggest that when the fibrils are formed, the structural content is similar regardless of when the rotenone is introduced (Figure 4 and Figure 5). The signal of the supernatant samples displays structural content characteristic of aggregated proteins containing substantial amount of β-sheet structure (Figure 4 and Figure 5). The signal intensity for the supernatant samples decreased when rotenone was introduced at later times during the fibrillation process. The lower signal intensity and similar structural content in the supernatant sample spectra indicate that at the time of the rotenone addition, a portion of the misfolded α-synuclein had aggregated and folded into structures that would not fold into fibrils. This supports the hypothesis that alternate paths to form aggregated species with β-sheet content take place during the fibrillation process. These results are also supported by the ThT kinetic assay (Figure 1), which showed that when rotenone was introduced at later times during the fibrillation process, the lag time of the fibrillating process did not decrease. The FTIR signals of the supernatant samples at the time of rotenone addition and at the end of the fibrillation process clearly show structural differences. The misfolded species in the supernatant at the time of rotenone addition were indistinguishable, and as mentioned above, are species that display high degree of random coil (Figure 2 and Figure 3). However, the supernatant samples at the end of the fibrillation process displayed secondary structural characteristics of aggregated species with increased β-sheet content (Figure 4 and Figure 5). The later supernatant structures are most likely the result of the formation of stable large prefibrillar species [40,107,108].

#### 2.2.3. Secondary Structure Assessment

The Amide I band can be used to evaluate the amount of secondary structure content present in a given sample through a combination of second derivative and FSD procedures [90,91,109,110,111]. This process establishes the presence of individual secondary structural components in a given sample. This analysis provides a band position unique to an individual secondary structural component and its contribution to the overall spectrum can be established. When the FSD and second derivative signals are congruent, the bands are used to extract the positions (cm^−1^) of the maxima, which can be used to curve-fit the data with a high degree of accuracy. Subsequently, the deconvoluted data are used to assign secondary structure components using empirically based data sets [90,91,112]. 

Deconvolution was applied to the spectra of the supernatants and precipitated samples at the time of rotenone addition during the fibrillation process and at the end of the fibrillation process. Figure 2 and Figure 4 show the original spectra, curve-fitted spectra, and deconvoluted signals. The secondary structural analysis is summarized in Table 2 and Table 3.

**Table 2:** ATR-FTIR evaluation of secondary structure content during the fibrillation process for human α-synuclein with rotenone introduced at selected time intervals.

Supernatant α-synuclein samples														
**Time (hours) ^a^**	0		15-min			30-min			1.0 h			3.0 h		
	Wave-number		%	Wave-number	%	Wave-number		%	Wave-number		%	Wave-number		%
**Structural assignment**	cm^−1^			cm^−1^		cm^−1^			cm^−1^			cm^−1^		
Turn or β-sheet	1692		3	1692	2	1695		4	1688		5	1687		3
Turn	1674		21	1682	7	1674		18	1673		20	1672		21
Loops/Disorder	1655		35	1664	44	1658		22	1661		15	1650		63
Disorder/Extended	1637		41	1639	47	1639		56	1643		61	1634		13
β-sheet														
Side chains														
**Precipitate α-synuclein samples**														
**Time (hours) ^a^**		0	15-min			30-min			1.0 h			3.0 h		
		Wave-number	%	Wave-number	%	Wave-number	%		Wave-number	%		Wave-number	%	
**Structural assignment**		cm^−1^		cm^−1^		cm^−1^			cm^−1^			cm^−1^		
Turn or β-sheet		1674	19						1676	3				
Turn				1674	28	1675	49		1674	27		1672	39	
Loops/Disorder		1650	60	1655	30	1654	25		1656	13		1655	10	
Disorder/Extended		1637	14	1639	31	1639	4		1642	47		1643	20	
β-sheet												1628	13	
Side chains		1610	7	1610	12	1610	23		1610	11		1610	8	

^a^ indicates the time of rotenone addition.

**Table 3:** ATR-FTIR-based evaluation of secondary structure content during the fibrillation process for human α-synuclein with rotenone introduced at selected time intervals and samples taken at the end of the fibrillation process.

Supernatant α-synuclein samples													
**Time (hours) ^a^**	0			15-min		30-min		1.0 h					
	Wave-number	%		Wave-number	%	Wave-number	%	Wave-number	%	Wave-number		%
**Structural assignment**	cm^−1^			cm^−1^		cm^−1^		cm^−1^		cm^−1^		
Turn or β-sheet	1695	1		1695	1	1685	7	1692	1	1692&1683		1&9
Turn	1974	18		1681	8	1671	15	1672	23	1671		17
Loops/Disorder	1660	38		1660	52	1657	13	1656	26	1657		27
Disorder/Extended	1643	24		1652 &1642	11&13	1652	44	1639	30	1642		20
β-sheet	1626	19		1625	24	1628	19	1626	16	1626		26
Side chains						1624	3	1613	4			
**Supernatant α-synuclein samples**													
**Time (hours) ^a^**	0			15-min		30-min		1.0 h					
	Wave-number	%		Wave-number	%	Wave-number	%	Wave-number	%	Wave-number		%
**Structural assignment**	cm^−1^			cm^−1^		cm^−1^		cm^−1^		cm^−1^		
Turn or β-sheet				1695	1	1693	0.5	1678	11	1690		1
Turn	1674	14		1682	1	1680	6	1668	8	1684		3
Loops/Disorder	1660	19		1664	31	1665	20	1655	18	1658		20
Disorder/Extended	1649	14		1642	23	1647	20	1644	6	1644		22
β-sheet	1631	46		1629	34	1629	46	1629	51	1626		48
Side chains	1616	8		1616	10	1616	9	1613	6	1616		6

^a^ indicates the time of rotenone addition.

### 2.3. Analyzing the Effect Rotenone on Bovine Serum Albumin by ATR-FTIR

To clarify the effect of rotenone on the α-synuclein FTIR signal in the low 1600 cm^−1^ range, bovine serum albumin (BSA) was monitored in the presence of 0 to 5 molar equivalents of rotenone. In this study, rotenone was introduced at the onset of the reaction and the ATR-FTIR analysis conducted after a 30-min incubation period. The BSA ATR-FTIR analysis was used to substantiate the findings of the α-synuclein-rotenone studies and to establish that the initial band observed in the low 1600 cm^−1^ region was a result of side chain interactions. The results of these analyses are shown in Figure 6. The deconvoluted ATR-FTIR signals of the BSA-rotenone after 30-min incubation clearly show a signal enhancement in the 1605–1620 cm^−1^ region. This effect, which is better observed in the superimposed second derivative signal (Figure 7), is associated with the side chain interactions [105,106]. The BSA misfolding can be interpreted as an event triggered by BSA interacting with the hydrophobic rotenone, resulting in lower stability of the native state of the protein. The BSA-rotenone interaction was monitored during the early stages of the pesticide exposure to show that the initial misfolding events did take place as evidenced by specific spectral changes arising from the side chain interactions. Similar events have previously been observed where the fibrillation was triggered by the formation of partially folded intermediate structures or by molten globule structures [40]. In studies with globular proteins, it has been established that partial unfolding is an imperative event for fibrillation to take place [113,114]. During the early stages of misfolding, long-range side-chain interactions are most likely responsible for the misfolding. Once the strands are stabilized by side-chain interactions, the transformation into the β-conformation begins to take place [115].

**Figure 8:**
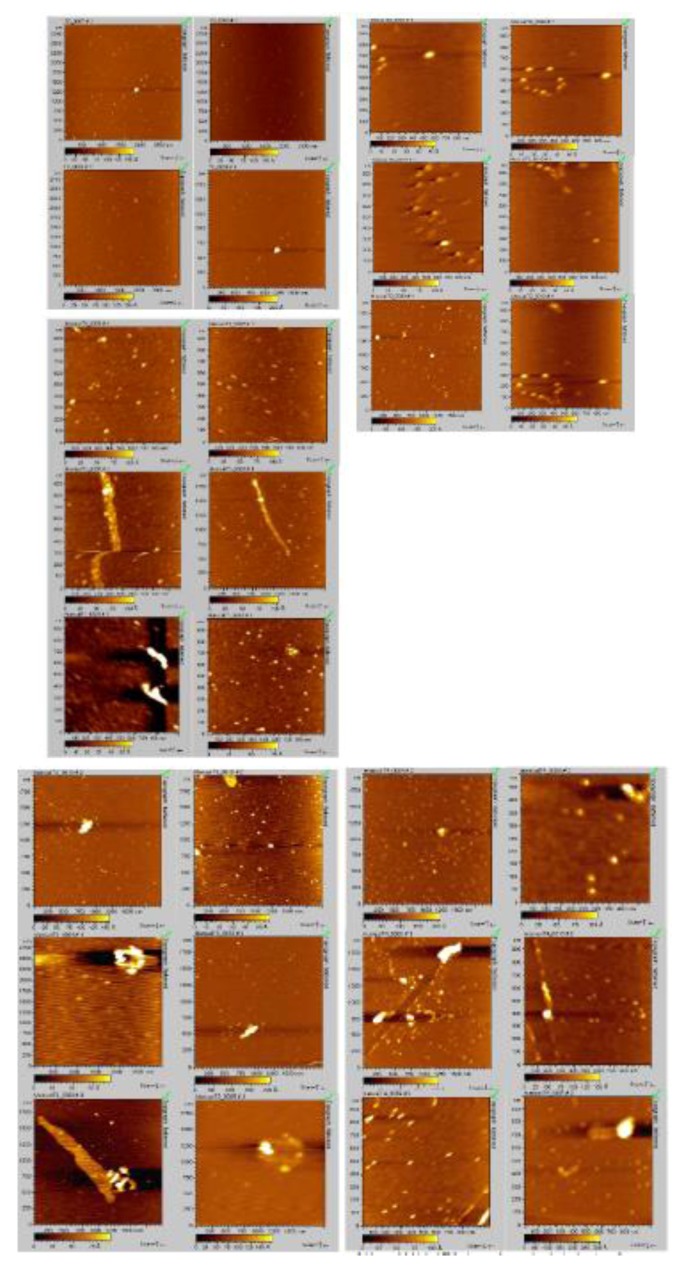
AFM micrographs obtained at time of rotenone addition: four images, upper left corner, 0 h; six images, upper right corner, 15 min, right; six images, middle left side, 30 min; six images, bottom left corner, 1 h; six images, bottom right corner, 3 h.

### 2.4. Analyzing the Morphologies of the α-Synuclein Aggregated Species by TEM and AFM

**Figure 9:**
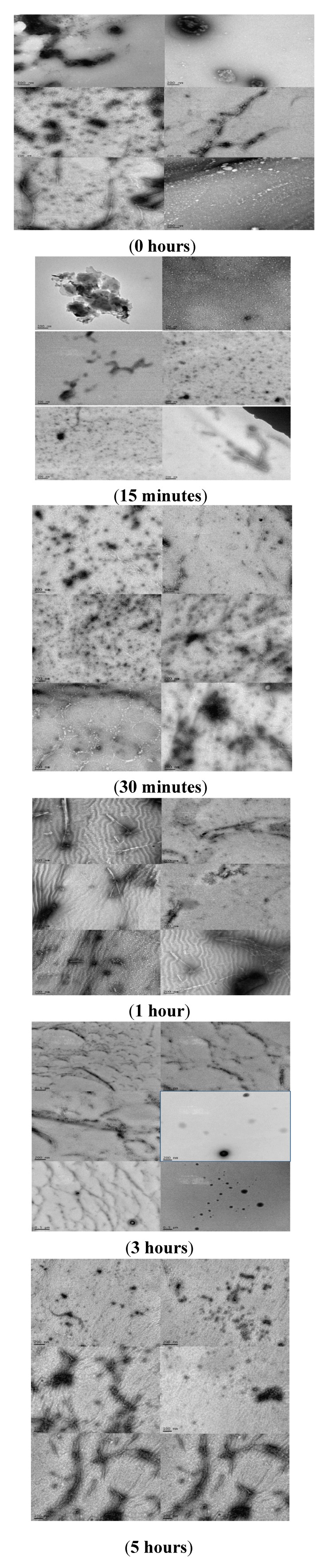
Multiple TEM micrographs, samples obtained at time of rotenone addition: 0 h; 15 min; 30 min; 1 h; 3 h; 5 h.

The effects of rotenone on the morphology of α-synuclein aggregates formed during the fibrillation process modulated by the addition of a pesticide at various times was characterized using TEM and AFM (Figure 8 and Figure 9). AFM and TEM micrograph analysis during these early stages of the fibrillation process shows that three major populations dominate in the samples. The first two populations consist of small and large aggregated species with significant size variance, and the third population consists of protofibrillar forms, characterized as long thin elongated species. The introduction of rotenone at selected time points promoted the formation of a mixed population of the various aggregated α-synuclein forms, which consisted of a dominant group of small aggregates and the presence of a smaller group of a significantly larger (~five times larger) aggregated species. The AFM and TEM micrographs clearly show differences in the size of the aggregated species. These effects were more pronounced when rotenone was introduced at earlier times during the fibrillation process (less than 60 min). Rotenone introduction when aggregated species were present appeared to be a tipping point for inducing the formation of the protofibrillar species. TEM analysis indicated that the protofibril samples were heterogeneous in length with average height of 4–7 nm (Figure 8). These results are consistent with previously published data [80,116,117,118] and suggest that α-synuclein present in a partially misfolded state associates more easily with pesticides; this association is most likely triggered by the hydrophobic character of the pesticide [119]. These effects are clearly observed in the TEM micrographs at times ranging from 0.25 to 1.0 h. During these early time points of rotenone introduction, the population of protofibrils was comparable with the populations of other aggregated species. However, with delayed rotenone introduction (three and five hours), the population of aggregated species dominated and the amount of protofibrils decreased significantly. These results are consistent with the presence of different size aggregated species and protofibrils in studies of α-synuclein aggregation in the presence of metals such as zinc, lead and copper [80,117,119].

## 3. Experimental Section

### 3.1. Materials

#### 3.1.1. Commercial Materials

Rotenone, bovine serum albumin (BSA) and thioflavine T of high purity grade were obtained from (Sigma-Aldrich Chemical Co., St. Louis, MO, USA) and used without further purification. All other chemicals used were of analytical grade from (Fisher Chemicals, Fairlawn, NJ, USA or Sigma Aldrich, St. Louis, MO, USA). 

#### 3.1.2. α-Synuclein Expression and Purification

Recombinant human wild type (wt) α-synuclein expression was achieved using *Escherichia coli* (*E*. *coli*) BL21 (DE3) cells transfected with pRK172/α-synuclein (wild type) wt plasmid (an invaluable gift of R. Jakes and M. Goedert, MRC Cambridge, UK). 

The step-by-step protocol ofα-synuclein expression and purification is presented below. First, per liter of preparative culture, 10-mL of inoculum Luria broth (LB) (SIGMA-ALDRICH Inc., St. Louis, MO, USA), containing BL21 (DE3) cells was incubated overnight in the presence of 75 mg/mL of α-carboxy bensyl penicillin disodium salt (carbenicillin) (US Biological). Next, 75 mg/mL carbenicillin was added to a 1-L of LB media sterilized by autoclave and mixed gently. This was followed by the addition of the overnight-grown inoculum. The medium was placed in a shaker rotating at 250–300 rpm at 37 °C, and the UV absorbance was monitored at 500 nm. When the UV absorbance signal reached 0.9–1.0 absorbance units (a.u.), the α-synuclein expression was induced by the addition of 0.5 mM of isopropyl β-D-thiogalactopyranoside (IPTG) (Gold Bio Technology, Inc., St. Louis, MO, USA), and the shaking was continued at the same speed for a period of five hours. Subsequently, the cells were collected through centrifugation at 4000 rpm for 20 min at 4 °C. It is important to point out that at this stage of protein expression and purification, cells could be stored at −20 °C for later purification or the purification process continued. From this point on, all purification steps proceeded under the ice-bath conditions, and buffers were kept or equilibrated to the ice-bath temperatures. The resultant pellet from 1-L culture was re-suspended in approximately 40 mL of lysis buffer consisting of 50 mM NaCl, 20 mM Tris-HCl, 0.10% Triton-X100, 0.20 mM phenylmethyl sulfonylfluoride (PMSF) pH 7.5 in a 0 °C ice bath. Cells were lysed through probe sonication for 3.5-minutes using 30-second sonication bursts with 40-second rests. The resultant suspension became more transparent and less viscous as cells were lysed. This suspension was brought to 30% ammonium sulfate saturation followed by 15 min centrifugation at 13,000 rpm. The resultant pellet, which contained unwanted material, was stored to monitor the α-synuclein purification. The supernatant containing α-synuclein was further brought to 50% ammonium sulfate saturation and again centrifuged for 15 min at 13,000 rpm. The resultant α-synuclein containing pellet was re-suspended in 25-mL of 10 mM TRIS-HCl, pH 7.5, buffer. Next, the re-suspended pellet was placed in 6–8 kDa cutoff dialysis tubing, Spectrapore 1, and extensively dialyzed *versus* equilibrating buffer consisting of 50 mM NaCl, 20 mM TRIS-HCl, pH 7.5, at 4 °C. The dialyzed α-synuclein was removed from the dialysis tubing and centrifuged for 15 min at 13,000 rpm to precipitate denatured contaminants. The supernatant was loaded onto a DEAE-Sepharose column (2.5 × 14 cm) pre-equilibrated at 4 °C by the 50 mM NaCl, 20 mM Tris-HCl, pH 7.5, buffer. Upon loading the α-synuclein, 6.5 mL fraction samples were collected at a rate of 1 mL/min using an isocratic buffer elution step in 70 mL pre-equilibrating buffer. After the isocratic elution step, a 700 mL linear gradient elution step, 50 to 500 mM NaCl in 20 mM Tris-HCl buffer at pH 7.5, was introduced and 6.5 mL fractions continued to be collected at the same elution rate of 1 mL/min. At the end of the purification process, the presence of α-synuclein was monitored by the UV absorbance at 275 nm. The samples were and further characterized by Coomassie-stained sodium dodecyl sulfate polyacrylamide gel electrophoresis (SDS-PAGE). Fractions containing α-synuclein were pooled and dialyzed extensively at 4 °C *versus* deionized water. After dialysis, denatured and precipitated material was removed by 15 min of centrifugation at 4 °C and 13,000 rpm. The protein concentration was determined on the basis of the UV absorbance at 275 nm (A_275_). To further remove high molecular weight contaminants, the protein sample was concentrated in a 5 kD MWCO Amicon, and loaded onto a Sephacryl-S300 size-exclusion column (15 × 150 cm) (Pharmacia), pre-equilibrated in 25 mM (NH_4_)_2_CO_3_ buffer. Upon loading the protein sample, 6.5 mL fraction samples were collected at a flow rate of 0.3 mL/min. α-Synuclein containing fractions were identified by monitoring the UV absorbance at 275 nm, and by Coomassie-stained SDS-PAGE. Then, the molecular mass of the protein was determined by electron spray ionization mass spectrometry (ESI-MS). Fractions containing purified α-synuclein were pooled and lyophilized in small aliquots, which were stored at −80 °C until further use.

### 3.2. Methods

#### 3.2.1. Sample Preparation for Aggregation Studies

At the time of use, 2–5 mg of lyophilized protein sample were dissolved in double deionized water adjusted to pH 10 ± 0.5 with 100 mM NaOH (about 1 mM final concentration) to solubilize any aggregated protein. The dissolved protein was incubated for 10min at room temperature. Afterwards, the protein sample was centrifuged for 30 min at 75,000 rpm in an air-driven ultracentrifuge (Beckman^®^ Airfuge) to remove large aggregates. The supernatant was carefully extracted, and the pH was adjusted to pH 7.4 with 1mM HCl and further addition of Tris-HCl buffer, pH 7.4. The protein purity was monitored by SDS-PAGE, fluorescence emission, and ESI-MS, while the protein concentration of 1–2 mg/mL was determined by measuring the UV absorbance at 275 nm using tyrosine’s extinction coefficient 0.40 mg^−1^ cm^2^.

Thioflavine T (ThT) was prepared as a 1 mM stock solution in nanopure water, filtered through a 0.2 mm sterile syringe filter, light protected to prevent oxidation, and stored at 4 °C until time of use. The ThT concentration was calculated by measuring absorbance at 420 nm and using an experimentally determined and documented extinction coefficient value of 24,420 M^−1^ cm^−1^ [120,121].

#### 3.2.2. Plate-Based Assay of α-Synuclein Aggregation in the Presence of Rotenone

*In vitro* rotenone effects on α-synuclein were monitored through the thioflavine T (ThT) assay. ThT assay samples consisted of 1 mg/mL, 0.5 mg/mL or 0.15 mg/mL (70 μM, 35 μM, 10 μM) of α-synuclein in buffer consisting of 20 mM Tris-HCl, pH 7.5/100 mM NaCl/20 μM ThT, 0.02% *v/v* NaN_3_. In a set of experiments, pesticides were present at sub-molar, equimolar or in excess amounts. Samples were prepared in amounts to monitor a minimum of three to five replicates, where 120 μL aliquots were distributed in a 96-well microtiter plate (Corning Inc., Corning, NY, USA). Each well contained a 1/8-inch diameter Teflon sphere (McMaster-Carr, Los Angeles, CA, USA). The plates were sealed with Mylar plate sealers (Thermo Electron Corporation, Vantaa Finland) and loaded into a fluorescence plate reader (Fluoroskan Accent^®^ Thermo Electron Corp., Vantaa, Finland). The samples were incubated at 37 °C with continuous shaking at 600 rpm and 2-mm diameter rotation with bottom reading. It should be noted that shaking was used to accelerate and unify the fibrillation process [122]. The ThT fluorescence emission signal was monitored at 30 min intervals, with sampling for 100 milliseconds (ms) at 485 nm while exciting at 444-nm wavelength. The resulting data consisted of a fluorescence signal of ThT expressed in arbitrary units (a.u.) displayed over time (in hours). The data generated from each well were used to plot the ThT fluorescence signal as a function of time, and fitted to a sigmoidal curve equation by Nielsen *et al*. [47,123] using SigmaPlot. It should be emphasized that the ThT fluorescence signal in a given well does not reflect an accurate quantifiable measure of the net amount of fibrils present. In fact, a number of factors can affect the ThT signal: the presence of compounds that may compete for the same binding site as ThT, the presence of different forms of fibrils, or the presence of ThT quenching agents free in solution or in fibril-bound form. Therefore, as a result of all of these interfering factors, ThT signal fluorescence signal intensity is typically normalized to determine kinetic differences [124].

#### 3.2.3. Manual Assay for Detecting α-Synuclein Fibrils and Aggregates

The manual aggregation and fibrillation process was conducted as follows. The formation of the α-synuclein fibrils and aggregates was monitored through changes in the ThT fluorescence emission signal at 482 nm upon excitation at 450 nm. Fibrils and aggregates were monitored using a minimum of six 1-mL reaction replicate samples of 70 μM α-synuclein in 20 mM Tris-HCl buffer, pH 7.4, containing 100 mM NaCl and 0.02% NaN_3_. The samples were then placed in a glass vial, stirred with a Teflon mini-stir bar on a stirring plate (Corning Inc., Corning, NY, USA), and incubated at 37 °C. The fibrillation was monitored by extracting 5-μL aliquots from the reaction vial and placing it in a 1-mL quartz cuvette containing 50 mM Tris-HCl buffer, pH 7.5, with 20-μM ThT and 100 mM NaCl. The ThT emission was recorded from 465 to 510 nm after excitation at 450 nm using a FluoroMax-3 spectrofluorometer, (Jobin-Yvon Horiba Inc., Edison, NJ, USA) equipped with a 150-W xenon lamp. The ThT assay was monitored using a 1-cm excitation light path 1-mL quartz cuvettes (Hellma USA, Plainview, NY, USA).

#### 3.2.4. Control Studies: Preparation of BSA-Rotenone Samples

Bovine serum albumin (BSA) fibrillation was monitored using a minimum of six 1-mL reaction replicate samples of 66 μM (2 mg/mL) BSA in a 20 mM Tris-HCl buffer, pH 7.4, containing 100 mM NaCl and 0.02% NaN_3_. Samples were placed in glass vials, stirred with a mini-Teflon stir bar and incubated at 37 °C for 30min in the presence of 0 to 5 molar equivalents of rotenone. At the end of the incubation period, a 200 μL aliquot sample was removed and prepared for ATR-FTIR analysis as described below.

#### 3.2.5. Sample Preparation for ATR-FTIR Measurements

The samples used to monitor the effects of rotenone on α-synuclein misfolded species and aggregates were prepared as follows. At selected times intervals during the α-synuclein fibrillation process, rotenone was introduced at a concentration of 105 μM (1.5 molar equivalents) and the reaction mixture was incubated for 30 min. Subsequently, a 200-μL aliquot of the fibrillating sample was removed and centrifuged at 14,000 rpm for 75 min in an Eppendorf microfuge. The supernatant was removed and concentrated to a 20-μL volume using Ultracel YM-3 regenerated cellulose filters (Millipore Corporation, Bedford, MA, USA). The precipitate was washed once with the same buffer and resuspended to 20 μL in 20 mM Tris-HCl buffer, pH 7.4, in the presence of 100 mM NaCl. The BSA-rotenone protein samples were prepared in a similar manner.

#### 3.2.6. Protein-Rotenone ATR-FTIR Spectroscopy

ATR-FTIR spectra were recorded on a ThermoNicolet Nexus 670 Spectrometer (Nicolet Instrument Corporation, Madison, WI, USA) equipped with an MTC detector and an out-of-compartment germanium trapezoidal internal reflectance element (IRE). Samples for the ATR-FTIR analysis were prepared as hydrated thin films on the surface of the IRE as previously described [90,111]. Once a thin film had been prepared, 1024 interferograms at 1-cm^−1^-resolution were co-added to generate each spectrum. The spectra were deconvoluted to determine the percentage of secondary structure present using the software program GRAMS32^®^ v4.02 (Galactic Industries Corporation, Salem, NH, USA) and SigmaPlot^®^ (Systat Software, Inc., Point Richmond, CA, USA). The peak positions were identified as a routine operation by both second derivative and Fourier self deconvolution (FSD) procedures. These peak positions were used to curve-fit the ATR-FTIR spectra; the peak values were fixed within a range of ±1 cm^−1^, and peak widths were allowed a variance of 15–35 cm^−1^. Peak heights, widths, and percentage Lorentzian were allowed to vary within specified parameters until a solution converged to a minimum. The percentages of each secondary structure were estimated by converting each deconvolved peak to a percent of the total curve-fitted spectra. 

#### 3.2.7. Transmission Electron Microscopy

Transmission electron microscopy (TEM) was used to analyze the morphology of the aggregated species and fibrils of α-synuclein, extracted at selected times during the early fibrillation stage. Sample preparation for electron microscopy involved using a 25 μL fibril or an aggregate sample diluted 1:4 with double distilled (ddi) water to a final volume of 100 μM. The fibrillar or aggregated sample was subsequently centrifuged for 45-min at 14,000 rpm. After centrifugation, the supernatant was removed, and the precipitated sample was re-suspended to 100 μL with ddi water and centrifuged at 14,000 rpm for another 45 min. Next, the supernatant was removed and the final washed precipitate was re-suspended to 25 μL with ddi water. An 8-μL fibrils droplet was placed on glow-discharged 300-mesh copper grids with type B carbon-coated support films. The protein sample was allowed to dry for five minutes, and excess liquid was blot-dried with filter paper by gently touching along the edge of the grid. Subsequently, the fibrils were negatively stained with 2% (*w/v*) aqueous uranyl acetate. Transmission electron micrographs (TEM images) of samples were obtained using a JEOL JEM-100B transmission electron microscope operated with an accelerating voltage of 80 kV. Images were in general taken at the magnification of 75,000× or as specified in the text and were generated with Gatan Digital Micrograph Software (Gatan Inc., Pleasanton, CA, USA, v3.61). 

#### 3.2.8. Atomic Force Microscopy

Simultaneously to TEM sample preparation, samples for AFM analysis were prepared at selected time intervals. Samples were prepared on freshly cleaved mica substrates (Pelco^®^ Mica Sheet, Ted Pella Inc., Redding, CA, USA), by placing a 5-μL aliquot sample of 1:5 diluted fibrillated α-synuclein with 20 mM phosphate buffer, pH 7.5, and adding 5-μL of 1 M NaCl. The α-synuclein/NaCl sample on mica was held along the edge with tweezers and gently tilted several times to ensure formation of a homogeneous layer. Samples were allowed to incubate for a minimum of 4 h to overnight, followed by extensive rinsing with ddi water to remove all salt and unbound protein. After rinsing, samples were allowed to air-dry in a loosely covered Petri dish. AFM images were obtained using a PicoScan Plus microscope equipped with MAC mode (Molecular Imaging, Phoeniz, AZ, USA). To obtain images, ultrasharp NCS16/AlBS probes were used (Mikromasch, Lady's Island, SC, USA). The AFM process consisted of a force constant of 45 N/m and 170-kHz resonant frequency. Typical scan rates ranged from 0.5–1 lines per second and a driver current of 10 ± 5 Å. Width and height of the AFM image structures were obtained using PicoScan5 and SPIP software.

## 4. Conclusions

The ThT kinetic assay demonstrates that α-synuclein is altered in the presence of rotenone. The analysis shows that the interaction of α-synuclein with rotenone results in an accelerated fibrillation process, reflected by an increase in the ThT fluorescence emission resulting from increased β-structure content. TEM and AFM analysis demonstrated the presence of at least three aggregated and misfolded populations during the early stages of the α-synuclein-rotenone fibrillating process: small aggregates, significantly larger aggregated species and various protofibrillar structures heterogeneous in length. 

The ATR-FTIR analysis shows that structural changes take place during the initial phases of the fibrillation process. These structural changes in the precipitated samples during the fibrillation process are reflected by an increasing signal in the 1610–1615 cm^−1^ range and are the result of the initial misfolding resulting from specific side-chain interactions. Furthermore, the structural character of the fibrils in the precipitate samples in the absence and presence of rotenone at the end of the fibrillation process is indistinguishable from sample to sample. In contrast, the supernatant samples analyzed during and at the end of the fibrillation process showed differences in the secondary structure character of the misfolded forms. The ATR-FTIR spectra of the supernatant samples at the end of the fibrillating process are consistent with the aggregated samples containing ~15–27% β-structure, compared with ~2–5% β-structure in the supernatant signals during the fibrillating process. The overall supernatant disordered structural content, resulting from random coil and unstructured content, decreased from ~62% present at the onset of the fibrillation process to ~47% by three hours into the fibrillating process and ~40–45% by the end of the fibrillation process. In the precipitate samples, the amount of β-sheet content increased from ~3–13% during the early phase of the fibrillating process to ~60% at the end of the fibrillation process. Throughout the fibrillation process the FTIR bands displayed the same signal profile with differences resulting only from the intensity, indicating that the amount of aggregated species is different, but the relative secondary structure generally remains the same. 

In summary, our work clearly shows that rotenone significantly affects the α-synuclein fibrillation process as evidenced by specific alterations in the kinetic ThT assay and the ATR-FTIR spectra as well as morphological differences observed by TEM and AFM techniques.
